# Apremilast ameliorates acute respiratory distress syndrome by inhibiting neutrophil-induced oxidative stress

**DOI:** 10.1016/j.bj.2022.09.001

**Published:** 2022-09-11

**Authors:** Yung-Fong Tsai, Chun-Yu Chen, Shun-Chin Yang, Yu-Ting Syu, Tsong-Long Hwang

**Affiliations:** a Graduate Institute of Natural Products, College of Medicine, Chang Gung University, Taoyuan, Taiwan; b Department of Anesthesiology, Chang Gung Memorial Hospital at Taoyuan, Taoyuan, Taiwan; c Graduate Institute of Clinical Medical Sciences, College of Medicine, Chang Gung University, Taoyuan, Taiwan; d Department of Anesthesiology, Taipei Veterans General Hospital and National Yang-Ming University, Taipei, Taiwan; e Research Center for Chinese Herbal Medicine and Graduate Institute of Health Industry Technology, College of Human Ecology, Chang Gung University of Science and Technology, Taoyuan, Taiwan; f Department of Chemical Engineering, Ming Chi University of Technology, New Taipei, Taiwan

**Keywords:** Apremilast, ARDS, Neutrophil, Oxidative stress, PDE4 inhibitor

## Abstract

**Background:**

The pathogenesis of acute respiratory distress syndrome (ARDS) is attributed to the dysregulation of oxidative stress and neutrophil recruitment. We aimed to investigate the anti-inflammatory effects of apremilast on human neutrophils and assess its efficacy for treating ARDS.

**Methods:**

We analysed superoxide anion generation, integrin expression, and adhesion in activated human neutrophils using spectrophotometry, flow cytometry, and immunofluorescence microscopy. Phosphorylation of extracellular signal-regulated kinase (ERK) and c-Jun N-terminal kinase (JNK) was determined using immunoblotting. A murine lipopolysaccharide (LPS)-induced ARDS model was used to evaluate the therapeutic effects of apremilast**.**

**Results:**

Apremilast significantly decreased superoxide anion production, reactive oxygen species (ROS) generation, cluster of differentiation (CD)11 b expression, and neutrophil adhesion in formyl-l-methionyl-l-leucyl-l-phenylalanine activated human neutrophils. Apremilast elevated cyclic 3′,5′-adenosine monophosphate (cAMP) and protein kinase A (PKA) activity in activated neutrophils. It reduced cellular cAMP-specific phosphodiesterase (PDE) activity and selectively inhibited enzymatic PDE4 activity. The activated cAMP/PKA pathway suppressed the phosphorylation of ERK and JNK as well as Ca^2+^ mobilization in activated neutrophils. All inhibitory effects of apremilast on activated neutrophils were reversed by a PKA inhibitor. *In vivo* examinations indicated that apremilast alleviated lung neutrophil infiltration, myeloperoxidase activity, pulmonary oedema, and alveolar damage in LPS-induced ARDS.

**Conclusion:**

Apremilast inhibits inflammatory responses after neutrophil activation via cAMP/PKA-dependent inhibition of ERK and JNK activation. Our study revealed apremilast suppresses oxidative stress and chemotaxis by selectively inhibiting PDE4 in neutrophils and thus protects against endotoxin-induced ARDS in mice. Apremilast can be used as an alternative off-label drug in treating acute lung damage.

## Graphical abstract

**Figure:**
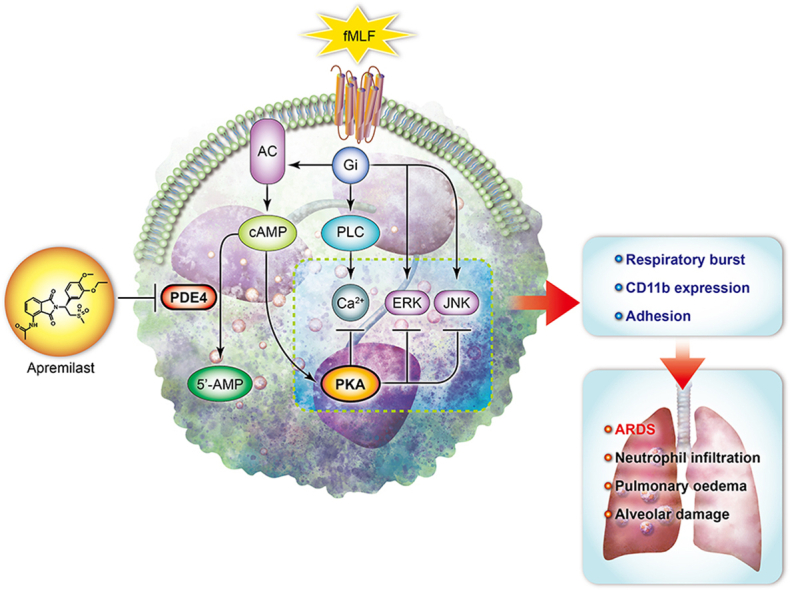

The coronavirus disease (COVID-19) pandemic has caused a surge in the number of patients with severe acute respiratory distress syndrome (ARDS) worldwide [1]. ARDS is a fatal respiratory failure with high morbidity and mortality rates [2,3]. It is triggered by multiple factors, including bacterial or viral infection, blood transfusion, chemical inhalation, sepsis, and trauma. The hallmarks of ARDS are hypoxaemia, excessive pulmonary inflammation, neutrophil adhesion and infiltration, alveolar barrier disruption, parenchymal and endothelial damage, and interstitial oedema [4]. Currently, anti-infection strategies and supportive care are mainstays for the treatment of ARDS, but the mortality rate remains high [5].

Neutrophils play a central role in the pathogenesis of ARDS and drive inflammatory milieu [6]. Excessive neutrophil activation and infiltration in the lungs are key features of ARDS and thus induce the disruption of the alveolar-capillary barrier and impairment of gas exchange in inflamed lungs [7]. Increased neutrophil infiltration is related to the severity and mortality in ARDS [6]. The production of superoxide anion and reactive oxygen species (ROS) contributes to oxidative stress when neutrophils are activated [8]. The dysregulated and overwhelming release of ROS derived from infiltrated neutrophils results in alveolar damage, recruitment of immune cells, the release of proinflammatory mediators, and interstitial tissue oedema [9]. Recruitment of large numbers of neutrophils can induce inflammatory epithelial damage, and this is mediated by interactions with the endothelium for rolling and adhesion [10]. Ligation of cluster of differentiation molecule (CD)11 b/CD18 on the neutrophil membrane to surface adhesion molecules on the epithelium mediates neutrophil adhesion [11].

Cyclic adenosine monophosphate (cAMP) plays a pivotal role in regulating numerous inflammatory responses in innate immune cells [12]. Therefore, cAMP-specific phosphodiesterases (PDEs) regulate cAMP concentration, and compartmentalisation has become a target for treating various inflammatory diseases. ARDS and pulmonary injury correlated with neutrophil dysregulation can be treated with cAMP-inducing drugs [13,14]. In neutrophils, PDE4 is the major subtype of PDE and is implicated in the pathogenesis of inflammatory diseases [15,16]. An inhibitor of PDE4 promotes intracellular cAMP accumulation and increases protein kinase (PK) A activity and thus reduces oxidative stress and integrin expression in activated neutrophils [14,17,18]. A non-selective PDE inhibitor, theophylline, has been clinically used for the treatment of pulmonary inflammation for more than 7 decades, but it presents serious concerns, including low safety margins and complex drug interactions [19]. To date, only few PDE4 inhibitors have been developed for efficient clinical treatment of patients with inflammatory diseases.

Apremilast is an orally selective PDE4 inhibitor approved by the Food and Drug Administration (FDA) in 2014 and provides a novel approach for treating moderate to severe plaque psoriasis and psoriatic arthritis. Apremilast reduces the generation of pro-inflammatory and anti-inflammatory mediators, including tumour necrosis factor (TNF)-α, interferon-γ, interleukin (IL)-23, and IL-10, via PDE4 inhibition [20,21], thereby inhibiting the infiltration of immune cells and pathogenesis in inflamed tissues of the skin and joints [21,22]. Apremilast has been found to suppress the infiltration of T lymphocytes, natural killer cells, and myeloid dendritic cells in psoriatic skin and to reduce the expression of IL-12, IL-17, IL-22, and IL-23 [23]. However, the mechanisms and therapeutic effects of apremilast in the regulation of oxidative stress in neutrophils and in ARDS therapy remain elusive and are not well understood.

This study aimed to determine the suppressive effects of apremilast on the release of superoxide anions and reactive oxidants, CD11b expression, and neutrophilic adhesion in N-formyl-L-methionyl-L-leucyl-l-phenylalanine (fMLF)-activated human neutrophils. We further aimed to evaluate the therapeutic potential of apremilast for off-label use in patients with ARDS.

## Material and methods

### Reagents

The chemicals used in this study and the manufacturers are as follows: apremilast (C_22_H_24_N_2_O_7_S, MW: 460.5) from BioVision (Milpitas, CA, USA); 2-(4-Iodophenyl)-3-(4-nitrophenyl)-5-((2,4-disulfophenyl)-2H-tetrazolium monosodium salt (WST-1) from Dojindo Molecular Technologies (Kumamoto, Japan); *N*-[2-[[3-(4-bromophenyl)-2-propenyl] amino]ethyl]-5-isoquinolines-ulfonamide (H89) and phenylmethylsulfonyl fluoride (PMSF) from Calbiochem (La Jolla, CA, USA); dextran from MP Biomedicals (Irvine, CA, USA); Ficoll from GE Healthcare (Boston, MA, USA); Hank's balanced salts solution (HBSS) from Gibco (Co Dublin, Ireland); trypan blue from Biological Industries (Kibbutz Beit-Haemek, Israel); dihydrorhodamine 123 (DHR123), Fluo-3/AM, and Hoechst 33,342 from Molecular Probes (Boston, MA, USA); fluorescein isothiocyanate (FITC)-conjugated anti-CD11b from BD Biosciences (San Jose, CA, USA); antibodies against phospho-extracellular signal-regulated kinase (ERK), ERK, phospho-c-Jun N-terminal kinase (JNK), JNK, phospho-p38 mitogen-activated protein kinase (MAPK), and p38 MAPK from Cell Signaling (Beverly, MA, USA); antibodies for immunohistochemistry (IHC) against lymphocyte antigen 6 complex locus G6D (Ly6G) from BioLegend (San Diego, CA, USA); and myeloperoxidase (MPO) from Abcam (Cambridge, CB2 0AX, UK). All other pharmacologic agents were purchased from Sigma–Aldrich (St. Louis, MO, USA).

### Neutrophil purification

The study protocol was approved by the ethics committee of the Chang Gung Memorial Hospital (IRB no. 201601307A3 and 201902217A3C601), and the study design followed the principles of the Declaration of Helsinki 1975. All blood samples were obtained from healthy volunteers who provided signed informed consent. Neutrophils were purified following dextran sedimentation and gradient centrifugation in Ficoll-Hypaque solution, as previously described [18]. Neutrophils were incubated in HBSS and kept at 4 °C before the assay.

### Assay for extracellular superoxide anion generation

Superoxide dismutase (SOD)-inhibitable reduction of ferricytochrome *c* was used to analyse superoxide anion generation [24]. After supplementation of ferricytochrome *c* (0.6 mg mL^−1^), neutrophils (6 × 10^5^ cells mL^−1^) were treated with dimethyl sulfoxide (DMSO; 0.1%, as vehicle control) or apremilast (0.03–1 μM) at 37 °C for 5 min and were then activated by fMLF (0.1 μM) for another 10 min. Before activation by fMLF, neutrophils were primed with cytochalasin B (CB, 1 μg mL^−1^). For phorbol 12-myristate 13-acetate (PMA, 10 nM) activation, neutrophils (3 × 10^5^ Cells mL^-1^) were induced for 15 min without CB co-intubation. Superoxide anion generation was calculated by monitoring the changes in absorbance at 550 nm using a spectrophotometer (U-3010; Hitachi, Tokyo, Japan).

### Assay for intracellular ROS generation

Dihydrorhodamine 123 (DHR123)-labelled neutrophils were incubated with DMSO or apremilast (0.1, 0.3, and 1 μM) for 5 min and were then stimulated with fMLF for 5 min after priming with CB (0.5 μg mL^−1^); the ROS levels were measured by detecting intracellular fluorescence intensity using a flow cytometer (BD Accuri™ C6; BD Bioscience, California, USA) [24].

### Assay for total ROS release

The total levels of intracellular and extracellular ROS were determined using luminol-enhanced chemiluminescence. Neutrophils (6.6 × 10^5^ cells mL^−1^) were preincubated with horseradish peroxidase (6 U mL^−1^) and luminol (37.5 μM) for 5 min and were then treated with DMSO or apremilast (0.1, 0.3, and 1 μM) before activation by fMLF (0.1 μM). Chemiluminescence responses were measured using an Infinite F200 Pro microplate reader (Tecan, Männedorf, Switzerland).

### Analysis of superoxide anion scavenging ability

A cell-free xanthine/xanthine oxidase system was used to analyse the superoxide anion scavenging function of apremilast. Xanthine oxidase (0.02 U mL^−1^) was incubated with DMSO, apremilast (0.1, 0.3, and 1 μM), or SOD (20 U mL^−1^) for 10 min in Tris buffer (50 mM, pH 7.4) with WST-1 (0.3 mM) at 30 °C, followed by with xanthine (0.1 mM). Superoxide-mediated WST-1 reduction was detected using a spectrophotometer at 450 nm [25].

### Analysis of free radical scavenging ability

1,1-Diphenyl-2-picrylhydrazyl (DPPH) and 2,2-azino-bis (3-ethylbenzothiazoline-6-sulfonic acid) diammonium salt (ABTS) are stable, nitrogen-based free radicals used to detect antioxidant capacity [26]. DPPH buffer (100 μM in ethanol) and ABTS buffer (7 mM in 2.45 mM potassium persulfate) were incubated with DMSO, apremilast (1, 3, and 10 μM), or α-tocopherol (3, 15, and 30 μM) for 15 min at 25 °C; the absorbance changes were determined at 517 and 734 nm, respectively.

### Quantification of lactate dehydrogenase (LDH) release

Human neutrophils (6 × 10^5^ cells mL^−1^) were incubated with DMSO or apremilast for 15 min at 37 °C, and the supernatants were collected to detect LDH levels. The total LDH was obtained by treatment with Triton X-100 (0.1%) for 30 min. The LDH concentration was measured using a commercial kit (Promega, Madison, WI, USA).

### Detection of CD11b expression

Human neutrophils (2.5 × 10^6^ cells mL^−1^) were incubated with DMSO or apremilast (0.1, 0.3, and 1 μM) at 37 °C for 5 min and were then stimulated with fMLF (0.1 μM)/CB (0.5 μg mL^−1^) for 5 min. The neutrophils were resuspended in HBSS (80 μL) after centrifugation at 4 °C and were then co-incubated with FITC-conjugated mouse anti-human CD11b antibody (1 μg) for 90 min at 4 °C. The immunofluorescence intensity was determined using flow cytometry [27].

### Neutrophil adhesion study

Human neutrophils (1 × 10^6^ cells mL^−1^) were pre-stained with Hoechst 33,342 (1 ng mL^−1^) for 10 min. After centrifugation, neutrophils were incubated with DMSO or apremilast (0.1, 0.3, and 1 μM) for 4 min. Hoechst 33,342-labelled neutrophils were incubated with bEnd.3 endothelial cells and fMLF (0.1 μM) for 30 min, and then fixed with 4% paraformaldehyde. The number of neutrophils adhering to endothelial cells was quantified using an inverted microscope (IX81; Olympus, Japan) [27].

### Measurement of cyclic adenosine monophosphate (cAMP) concentration

Human neutrophils (5 × 10^6^ cells mL^−1^) were pre-treated with DMSO or apremilast (0.1, 0.3, and 1 μM) for 5 min before stimulation with fMLF (0.1 μM) for 1 min. The reaction was terminated by the addition of dodecyltrimethylammonium bromide (0.5%). After centrifugation at 3000 *× g* for 5 min, the supernatant was analysed for cAMP concentration [18]. An enzyme immunoassay kit was used to measure the cAMP concentration (Amersham Biosciences, Buckinghamshire, UK).

### Assay for protein kinase A(PKA) activity

Human neutrophils (2.5 × 10^7^ mL^−1^) were incubated with DMSO or apremilast (0.1, 0.3, and 1 μM) for 5 min before adding fMLF for 30 s at 37 °C. After centrifugation at 500*g* for 5 min at 4 °C, neutrophils were immersed and lysed in lysis buffer. The supernatants obtained were used for the protein kinase A (PKA) activity assay using a nonradioactive PKA kit (Arbour Assays, Michigan, USA).

### Detection of PDE activities

Neutrophils (5 × 10^7^ cells mL^−1^) were ruptured by sonication in lysis buffer [25 mM Tris-hydrochloride (HCl) (pH 7.4), 1 mM PMSF, 6 mM magnesium chloride (MgCl_2_), 0.25 M sucrose, 1% protease inhibitor cocktail, and 1% phosphatase inhibitor cocktail]. After centrifuging at 1400 *g* for 15 min, the cytosolic fraction was used as neutrophil-origin PDEs. For assaying the activities of PDEs, cytosolic fraction or commercial PDE enzymes (including PDE4A1, PDE4B2, PDE4C1, PDE4D2, PDE3B, and PDE7A) were co-incubated with the PDE assay buffer (50 mM Tris–HCl, pH 7.4, and 6 mM MgCl_2_) and test reagents for 10 min. Further, cAMP (60 nM) was added, and this reaction mixture was kept for 45 min before incubation with the fluorescence donor (anti-cAMP antibody) and fluorescence acceptor (Dye2-labelled cAMP) for 60 min (Cisbio International, Bagnol-sur-Ceze, France). The fluorescence changes were monitored using an Infinite F200 Pro microplate reader.

### Assay for adenylyl cyclase (AC)

Neutrophils (1.5 × 10^7^ cells mL^−1^) were sonicated in 4 °C lysis buffer [25 mM Tris–HCl (pH 7.4), 1 mM PMSF, 10 μM pepstatin, 10 μM leupeptin, 5 mM MgCl_2_, 0.25 M sucrose, and 2 mM ethylenediaminetetraacetic acid (EDTA)]. Unruptured cells were separated by centrifugation at 4 °C, and the supernatant was centrifuged once again at 18,000 *× g* for 20 min. The pellet was resuspended in a reaction mixture [25 mM Tris (pH 7.4), 15 mM MgCl_2_, 0.5 mM 3-isobutyl-1-methylxanthine (IBMX), 7.5 mM creatine phosphate, 3 units creatine phosphokinase, 0.5 mM dithiothreitol, and 1 mM adenosine triphosphate (ATP)] for 20 min at 30 °C. The reaction was stopped by boiling, and cAMP concentration was detected using an enzyme immunoassay kit (Amersham Biosciences, Buckinghamshire, UK).

### Immunoblotting assay

Human neutrophils were pre-treated with H89 (5 μM) for 5 min at 37 °C and then co-incubated with test reagents for another 5 min, followed by stimulation with or without fMLF (0.1 μM)/CB (0.5 μg mL^−1^) for 30 s. Neutrophils were then lysed with sample buffer [62.5 mM Tris–HCl (pH 6.8), 5% β-mercaptoethanol, 2% sodium dodecyl sulphate (SDS), 10% glycerol, 0.01% bromophenol blue, 1% protease inhibitor cocktail, and 1% phosphatase inhibitor cocktail]. Samples were obtained by centrifugation at 14,000 *× g* for 20 min at 4 °C. Denatured proteins were separated by electrophoresis on 12% SDS-polyacrylamide gel and blotted onto nitrocellulose membranes (Whatman, Perkin–Elmer Life Science). Target proteins were identified with the corresponding primary antibodies [protein kinase B (Akt), 1:1000; JNK, 1:1000; ERK, 1:3000; p38, 1:5000] and horseradish peroxidase-conjugated secondary antibodies (1:3000, 1:5000, 1:8000, and 1:8000, respectively). The signal intensities of bands were analysed using a densitometer (UVP, Upland, CA) by detecting peroxidase activity with an enhanced chemiluminescence system (Amersham Biosciences).

### Intracellular calcium concentration ([Ca^2+^]_i_) assay

Human neutrophils (3 × 10^6^ cells mL^−1^) were labelled with Fluo-3 AM (2 μM) at 37 °C for 30 min and were further treated with the test reagents before adding fMLF. The intracellular calcium concentration ([Ca^2+^]_i_) was measured using a spectrofluorometer (Hitachi F 4500; Tokyo, Japan) with excitation/emission wavelengths of 488/520 nm [14].

### Lipopolysaccharide (LPS)-induced acute respiratory distress syndrome (ARDS) in mice

Eight-week-old male BALB/c mice acquired from BioLASCO Taiwan Co., Ltd (Taipei, Taiwan) were used. All study protocols were approved by the Institutional Animal Care and Use Committee of the Chang Gung University. Mice were allocated to four groups as follows: sham-operated mice treated with vehicle and ARDS animals pre-treated with vehicle or apremilast at a dose of 5 or 10 mg kg^−1^ intravenously. The vehicle control was 0.5% (w/v) methylcellulose 400 dissolved in saline. After the administration of apremilast for 1 h, intratracheal instillation of 2 mg kg^−1^ LPS (*Escherichia coli* serotype 0111:B4) was carried out. After LPS induction for 5 h, the mice were sacrificed. The lung samples were excised for assaying MPO activity and for a histological examination [27].

### Histological examination

Lung samples were added to 10% formalin and embedded in 100% paraffin wax. For haematoxylin–eosin (H/E) staining, 5 μm-thick sections of the specimen were made. For immunohistochemical staining, the dewaxed sections were incubated with anti-Ly6G (BioLegend, CA, USA), anti-MPO (Abcam, Cambridge, UK), and anti-4-HNE (Bioss, MA, USA) antibodies. The morphology was visualised using a light microscope (Olympus Microscopy IX81). The pathological features of H/E-stained and IHC sections were quantitated using ImageJ software. The severity of pulmonary oedema was presented by quantitating the percentage of tissue area in H/E-stained sections.

### Determination of lung MPO activity

The lung samples were frozen at −70 °C and then soaked in phosphate-buffered saline (PBS) containing 0.5% hexadecyltrimethylammonium bromide before being homogenised by sonication. MPO activity was determined by a colorimetric assay using combined hydrogen peroxide (H_2_O_2_) and *o*-dianisidine HCl. Absorbance changes, represented as MPO activity, were monitored at 450 nm wavelength. MPO levels were referenced with a standard curve of commercial MPO (US Biological, MA, USA) and expressed as units per gram of lung specimen.

### Statistical data and analyses

The data and statistical analysis complied with the recommendations on experimental design and analysis in pharmacology [28]. All data are shown as box-and-whisker plots (median, min–max) or line plots [mean, standard error of mean (SEM)]. Statistical analysis was performed using a one- or two-way analysis of variance, followed by Tukey's multiple comparison test. All statistical analyses were performed using GraphPad Prism software (GraphPad Software, San Diego, CA, USA). Statistical significance was set at *p* < 0.05. Data are presented as mean ± SEM, and N values are independent experiments.

## Results

### Inhibition of oxidative stress

**Fig. 1:**
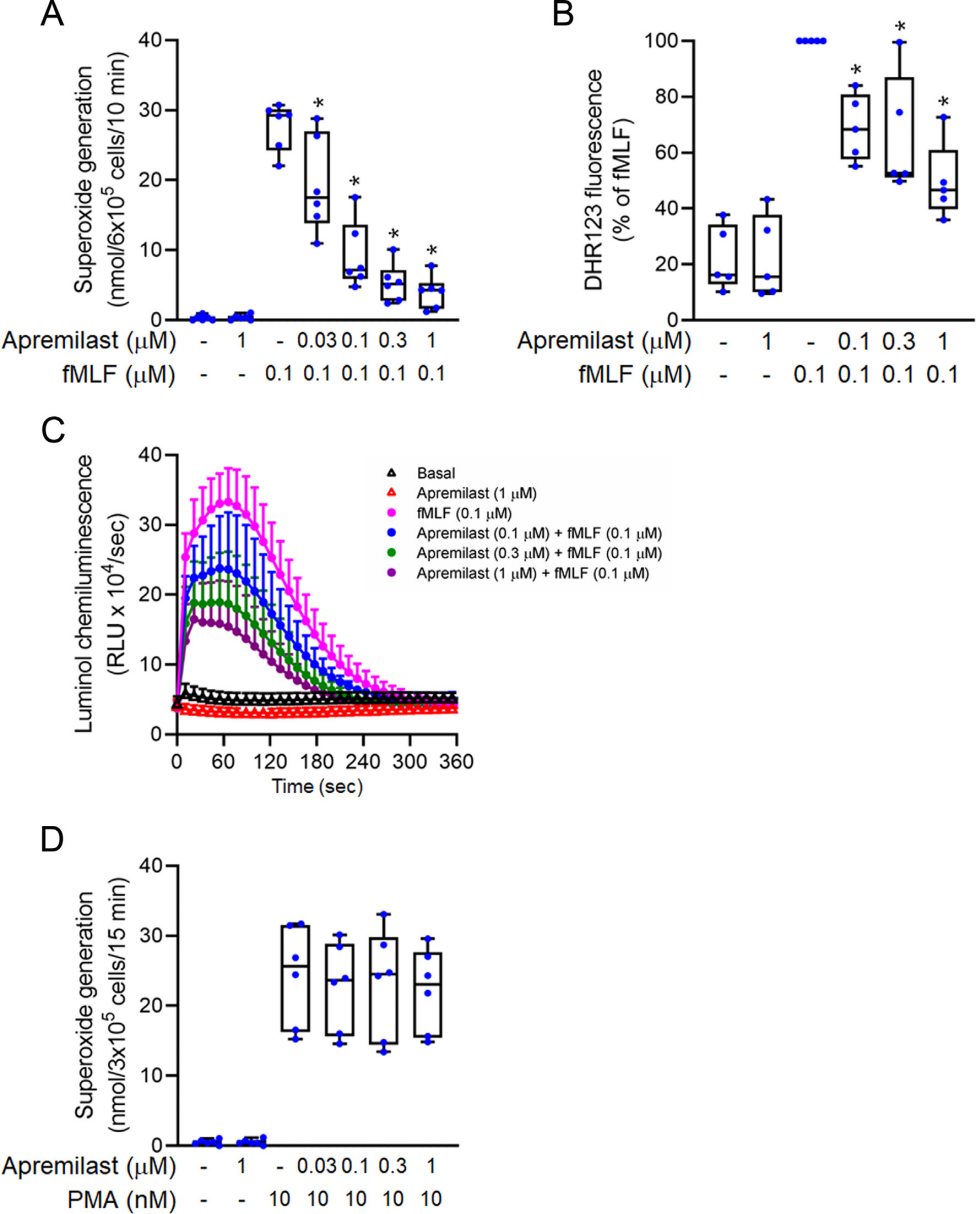
**Apremilast inhibits oxidative stress in activated human neutrophils**. (A) Human neutrophils were incubated with dimethyl sulfoxide (DMSO) or apremilast (0.03–1 μM) for 5 min before being stimulated with *N*-formyl-l-methionyl-l-leucyl-l-phenylalanine (fMLF) for an additional 10 min. Extracellular release of superoxide anion was measured by assay of superoxide dismutase (SOD)-inhibitable cytochrome *c* reduction using a spectrophotometer (n = 6) (B) Neutrophils were dyed with a dihydrorhodamine 123 (DHR123) probe, followed by dimethyl sulfoxide (DMSO) or apremilast (0.1–1 μM) treatment. Intracellular reactive oxygen species (ROS) levels were measured using flow cytometry after activation of fMLF (0.1 μM) (n = 5) (C) Intracellular ROS concentrations induced by fMLF were detected by a luminol–peroxidase assay (n = 5) (D) Human neutrophils were incubated with DMSO or apremilast (0.03–1 μM) for 5 min before being stimulated with phorbol 12-myristate 13-acetate (PMA) (10 nM) for 15 min. Extracellular release of superoxide anion was measured by assay of SOD-inhibitable cytochrome *c* reduction using a spectrophotometer (n = 6). Data represent the mean ± SEM from independent experiments. ∗*p* < 0.05 compared with the control value.

Superoxide anions and ROS are indicators of oxidative stress in activated human neutrophils. Apremilast (0.03–1 μM) significantly inhibited the release of extracellular superoxide anions in activated human neutrophils (Fig. 1A). The inhibitory effect occurred in a dose-dependent manner, and the value of the half-maximal inhibitory concentration (IC_50_) obtained was 0.06 ± 0.02 μM. At the ROS level, apremilast obviously reduced the activation of neutrophil-produced intracellular ROS (Fig. 1B) and total ROS (Fig. 1C), and the IC_50_ values were 0.22 ± 0.08 and 0.23 ± 0.07 μM, respectively. Apremilast did not alter the generation of superoxide anions and ROS in resting human neutrophils (Fig. 1). Furthermore, apremilast also failed to alter PMA-induced superoxide anion generation, suggesting apremilast didn't supress extracellular superoxide anion release from neutrophils by protein kinase C signaling pathways (Fig. 1D).

In addition, our results demonstrated that apremilast did not interfere with the balance of free radicals in WST-1, DPPH, and ABTS cell-free systems (Supplementary Fig. 1A–C), where SOD and α-tocopherol were used as positive controls. Cytotoxicity was expressed as neutrophil viability by LDH release. Even at a high dose of 1 μM, apremilast was not found to be toxic to neutrophils (Supplementary Fig. 1D).

### Inhibition of CD11b expression and adhesion ability of activated neutrophils

**Fig. 2:**
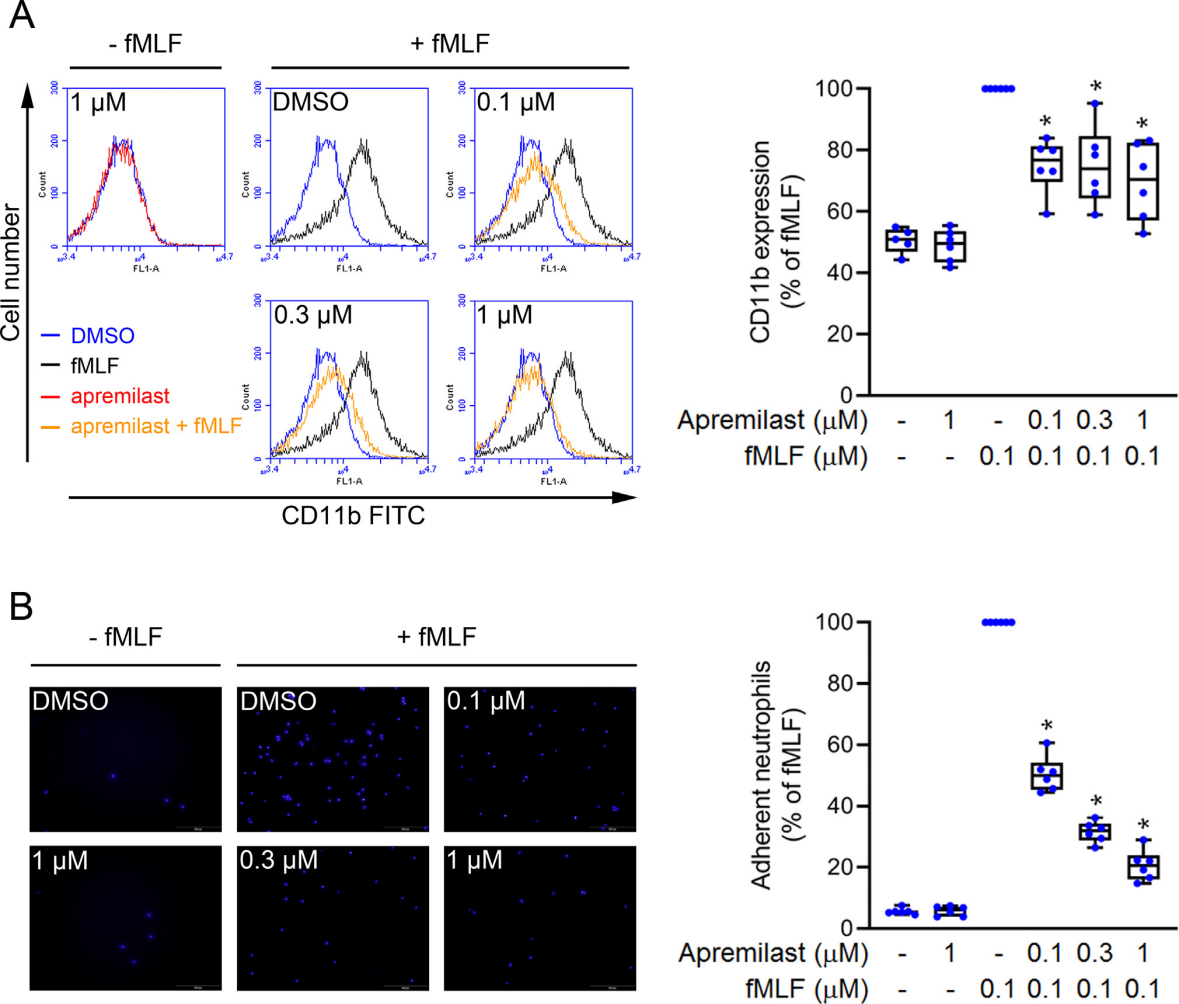
**Apremilast reduces CD11b expression and neutrophil adhesion**. (A) After neutrophils were pre-treated with dimethyl sulfoxide (DMSO) or apremilast (0.1, 0.3, and 1 μM), cells were stimulated with *N*-formyl-l-methionyl-l-leucyl-l-phenylalanine (fMLF) (0.1 μM) for 5 min. The fluorescence intensity of FITC-anti-CD11b in neutrophils was monitored as CD11b expression using a flow cytometer (n = 6) (B) Neutrophils were stained with Hoechst dye and then treated with DMSO or apremilast (0.1, 0.3, and 1 μM) before activation by fMLF (0.1 μM). Induced neutrophils were co-incubated with lipopolysaccharide (LPS)-treated endothelial cells for 15 min. Using a microscope, the counts of neutrophils adhering to endothelial cells were observed, and the data were summarised (n = 6). Data represent the mean ± SEM from independent experiments. ∗*p* < 0.05 compared with the control value.

Apremilast decreased the fMLF-stimulated expression of CD11b (Fig. 2A) and diminished neutrophil adhesion in a dose-dependent manner (Fig. 2B), and the IC_50_ values were 1.24 ± 0.58 and 0.09 ± 0.01 μM, respectively. The above data revealed that apremilast inhibited fMLF-induced neutrophil adhesion and chemotactic responses.

### Reducing cyclic adenosine monophosphate (cAMP)-specific PDE4 activity to enhance cAMP/PKA signaling

**Fig. 3:**
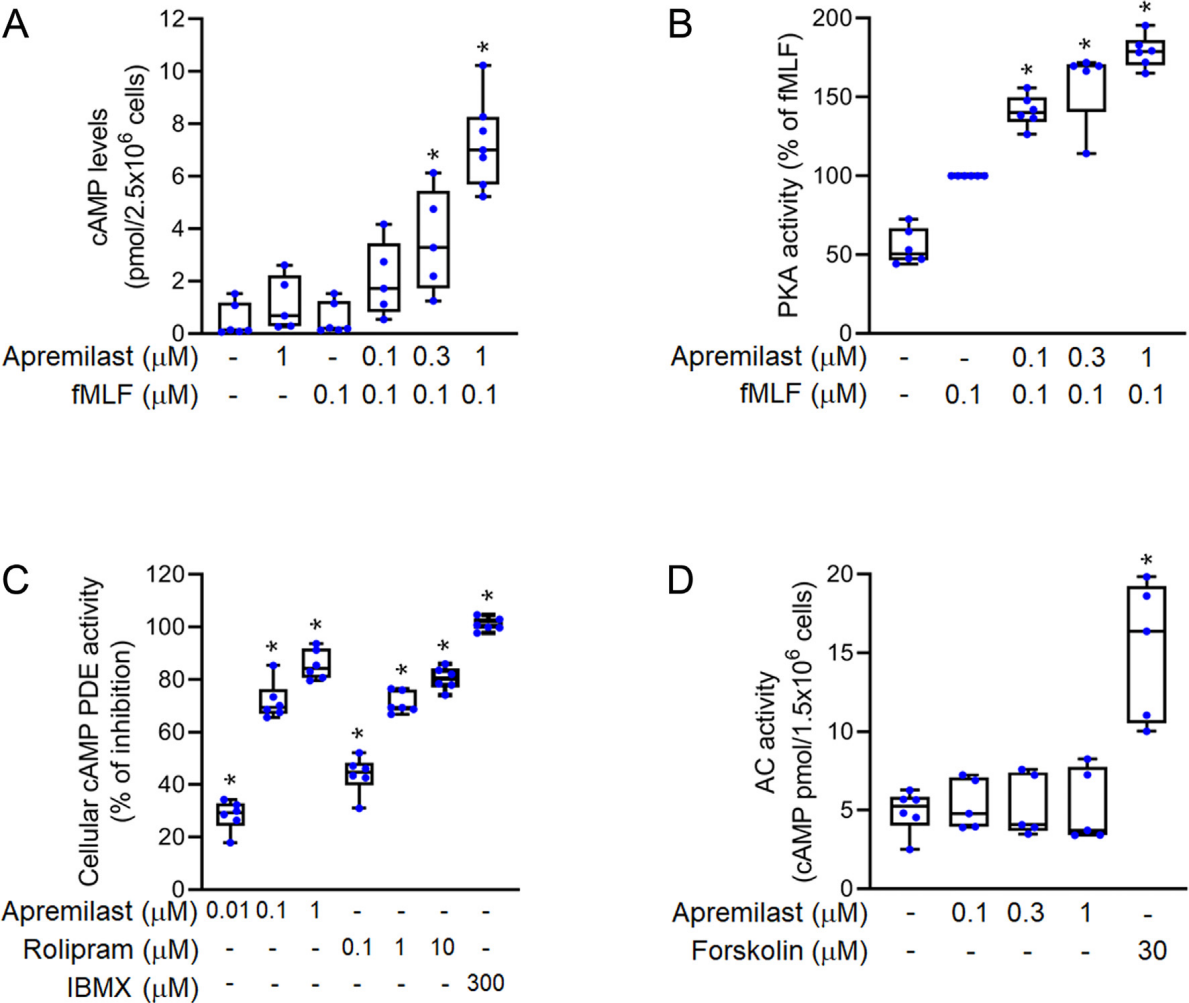
**Apremilast inhibits cyclic adenosine monophosphate (cAMP)-specific PDE4 activity to enhance cAMP/PKA signaling**. Neutrophils were incubated with dimethyl sulfoxide (DMSO) or apremilast (0.01–1 μM) and stimulated with *N*-formyl-l-methionyl-l-leucyl-l-phenylalanine (fMLF) (0.1 μM) for 1 min (A) cAMP levels and (B) PKA activities were assayed using enzyme immunoassay kits (n = 5–7) (C) For intracellular cAMP-specific PDE assay, cytosolic fractions from neutrophils were mixed with apremilast (0.01, 0.1, and 1 μM), rolipram (0.1, 1, and 10 μM), and 3-isobutyl-1-methylxanthine (IBMX, 300 μM). After the addition of the substrate cAMP (60 nM), PDE activity was analysed by the addition of each of the HTRF reagents (cAMP-d2 and anti-cAMP-cryptate). Enzymatic activities were measured using an HTRF reader (n = 6) (D) For the adenylyl cyclase (AC) activity assay, membrane fractions from neutrophils were mixed with apremilast (0.1, 0.3, and 1 μM) or forskolin (30 μM) with ATP (1 mM) for 20 min. The cAMP levels were determined using an enzyme immunoassay kit (n = 5). Data represent the mean ± SEM from independent experiments. ∗*p* < 0.05 compared with the control value.

Upregulation of the cAMP/PKA pathway can suppress superoxide anion formation, ROS production, and CD11b expression in neutrophils [18,29]. Our results showed that apremilast did not have a significant effect on cAMP levels in resting human neutrophils. However, in the presence of fMLF, apremilast (0.1, 0.3, and 1 μM) produced a synergistic increase in cAMP concentration in activated neutrophils (Fig. 3A). The PKA activity was found to increase due to apremilast in fMLF-activated neutrophils (Fig. 3B). We also tested the effects of apremilast on cellular PDE activity and found that it significantly inhibited cAMP-specific PDE activity in neutrophils (Fig. 3C) with an IC_50_ value of 0.03 ± 0.00 μM. The level of intracellular cAMP is the result of a balance between synthesis by ACs and degradation by PDEs. In this study, we found that apremilast did not interact with the AC activity of human neutrophils (Fig. 3D). The positive indicators used in the respective studies were rolipram (PDE4 inhibitor), forskolin (AC activator), and IBMX (non-selective PDE inhibitor).

### Selectivity in PDE4 inhibition

**Fig. 4:**
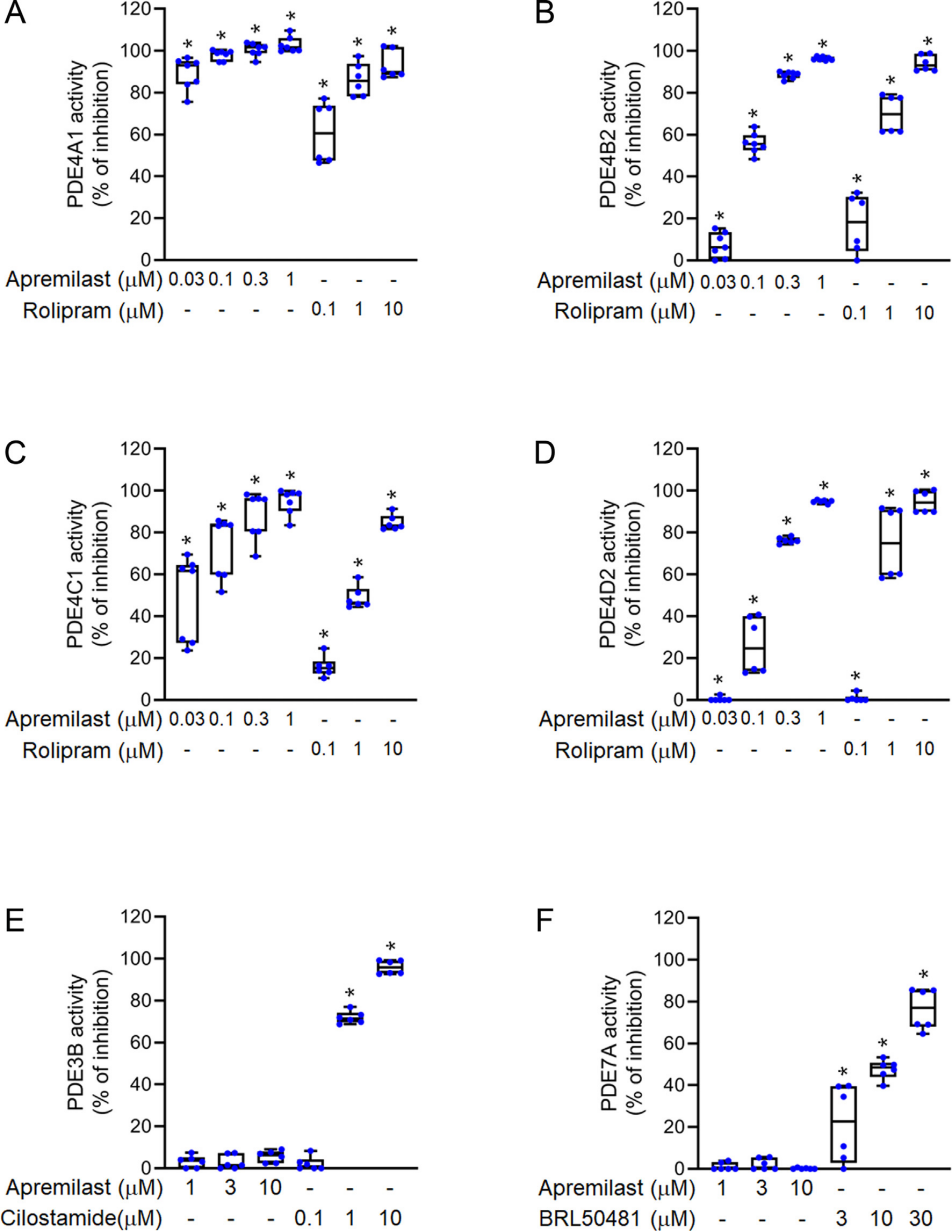
**Selectivity of apremilast for PDE4 inhibition**. To test the effects of apremilast on activities of PDE isoforms (A) PDE4A1 (B) PDE4B2 (C) PDE4C1 (D) PDE4D2 (E) PDE3B, or (F) PDE7A were incubated with the substrate, cyclic adenosine monophosphate (cAMP) (60 nM). dimethyl sulfoxide (DMSO) or apremilast (0.03–10 μM) was added to the mixture; further, HTRF reagents (anti-cAMP-cryptate and cAMP-d2) were added. Enzymatic activities were determined using an HTRF reader. Rolipram, cilostamide, and BRL50481 were the corresponding positive controls. Data represent the mean ± SEM from independent experiments (n = 6–7). ∗*p* < 0.05 compared with the control value.

The majority of cAMP-specific PDEs in human neutrophils are PDE3, PDE4, and PDE7 [30]. Apremilast showed selectivity in inhibiting PDE4 isozymes but failed to suppress PDE3 or PDE7 activity (Fig. 4). PDE4 isoforms include PDE4A, PDE4B, PDE4C, and PDE4D. The selectivity among individual PDE4 isoforms by apremilast was low. The IC_50_ values for the inhibition of PDE4A, PDE4B, PDE4C, and PDE4D2 activities by apremilast were 2.33 ± 0.71, 87.20 ± 4.01, 63.10 ± 23.30, and 164.30 ± 11.50 nM, respectively. Rolipram, cilostamide (PDE3 inhibitor), and BRL 50481 (PDE7 inhibitor) were used as positive controls.

### PKA signaling mediates neutrophil responses

**Fig. 5:**
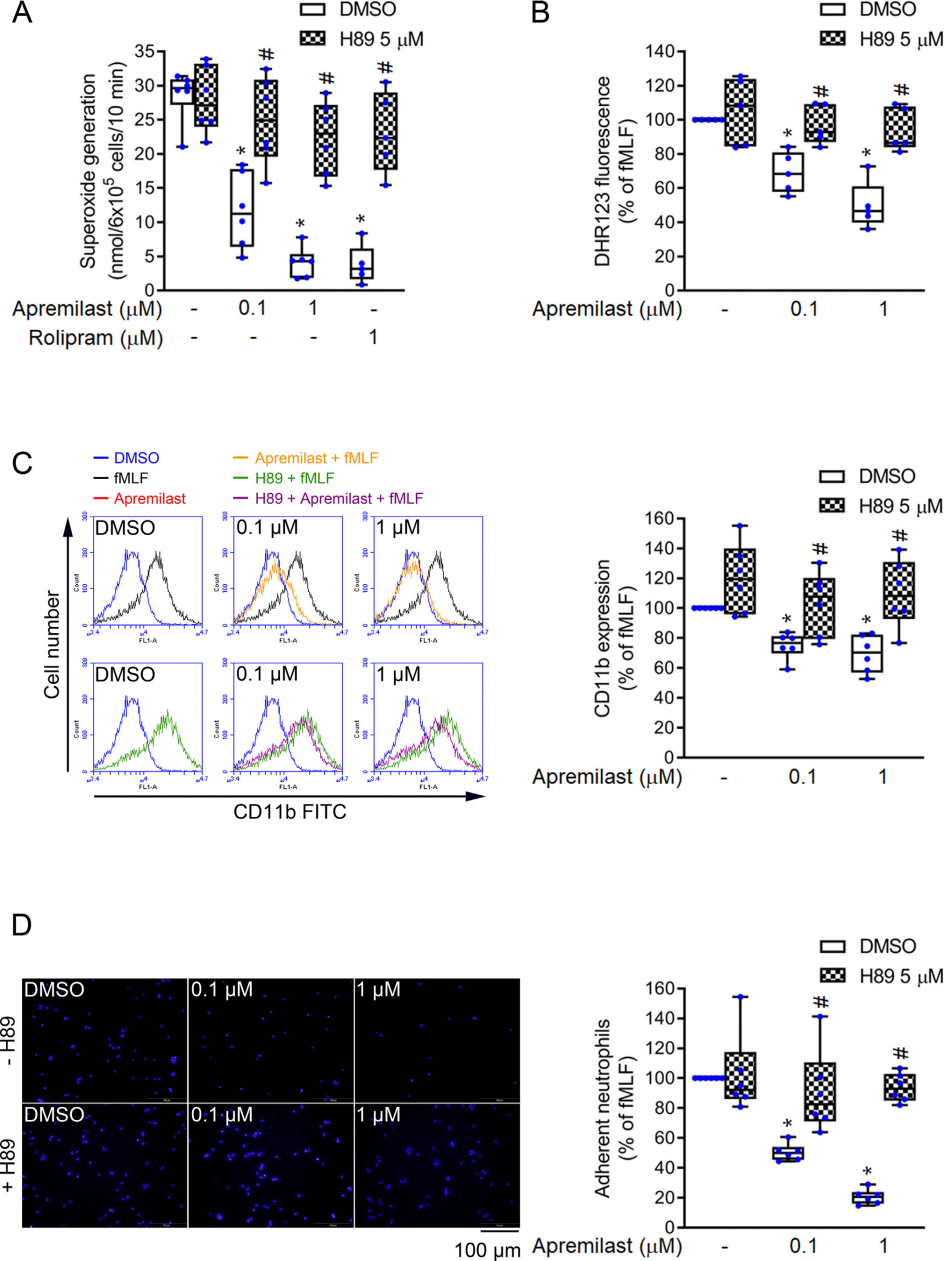
**Apremilast inhibits neutrophil responses mediated through PKA signaling**. N-[2-[[3-(4-bromophenyl)-2-propenyl] amino]ethyl]-5-isoquinolines-ulfonamide (H89) is a PKA inhibitor used to reverse neutrophil functions. After pre-treatment with H89 (5 μM) for 5 min, neutrophils were pre-treated with dimethyl sulfoxide (DMSO), apremilast (0.1 and 1 μM), or rolipram (1 μM). The inhibition of *N*-formyl-l-methionyl-l-leucyl-l-phenylalanine (fMLF)-induced neutrophil responses (A) superoxide anion release (n = 5–6) (B) intracellular ROS production (n = 5) (C) CD11b expression (n = 6), and (D) neutrophil adhesion (n = 6) were reversed by H89. Data represent the mean ± SEM from independent experiments. ∗*p* < 0.05 compared with control (fMLF only). ^#^*p* < 0.05 compared with its corresponding group (without PKA inhibitor).

Apremilast upregulated cAMP/PKA signaling in activated neutrophils. We further verified whether the neutrophilic inflammatory responses, including oxidative stress, CD11b expression, and cell adhesion, are mediated by PKA. When human neutrophils were pre-treated with the PKA inhibitor, H89, the inhibitory effects of apremilast on superoxide anion generation and ROS production were markedly abrogated in fMLF-activated neutrophils (Fig. 5A and B). The PDE4 inhibitor, rolipram, inhibited neutrophil superoxide anion release and was referenced as a positive indicator. This evidence supports the view that apremilast inhibits the oxidative stress increase in activated neutrophils mediated through cAMP/PKA signaling. In addition, our results confirmed that the inhibitory effects of apremilast on neutrophil CD11b expression and adhesion were also efficiently reversed by pre-treatment with H89 (Fig. 5C and D). These data indicate that apremilast inhibits oxidative stress increase, CD11b expression, and cell adhesion through the cAMP/PKA pathway.

### Intracellular calcium ([Ca^2+^]_i_) influx

**Fig. 6:**
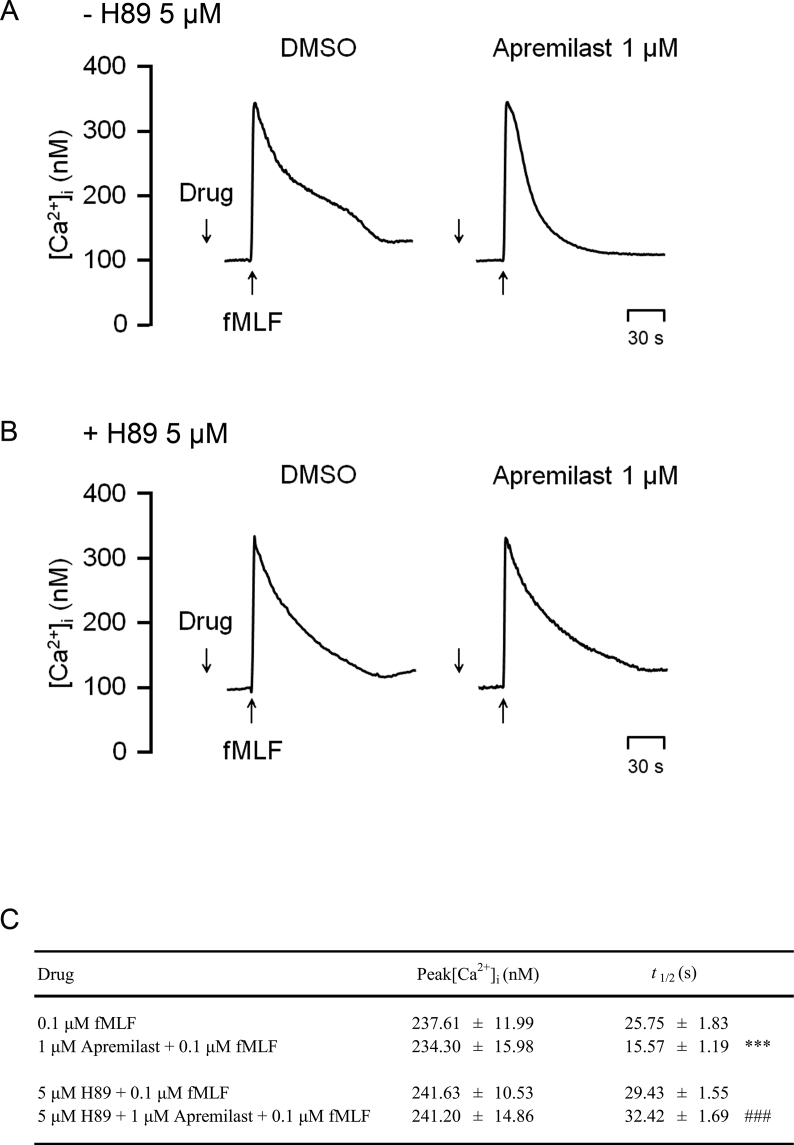
**Apremilast inhibits Ca^2+^ mobilization in activated neutrophils mediated by PKA signaling**. Fluo-3-dyed neutrophils were pre-treated with N-[2-[[3-(4-bromophenyl)-2-propenyl] amino]ethyl]-5-isoquinolines-ulfonamide (H89) (5 μM) for 5 min and then with dimethyl sulfoxide (DMSO) or apremilast (1 μM) for an additional 5 min. Human neutrophils were activated by *N*-formyl-l-methionyl-l-leucyl-l-phenylalanine (fMLF) (0.1 μM), and intracellular calcium concentration ([Ca^2+^]_i_)-time curves are presented. The peak [Ca^2+^]_i_ and *t*_*1/2*_ values were measured and listed as the mean ± SEM from independent experiments (n = 6). ∗*p* < 0.05 compared with the control value. ^#^*p* < 0.05 compared with its corresponding group (without H89 pre-treatment).

[Ca^2+^]_i_ mobilizaation plays an essential role in neutrophil activation [31]. As illustrated in Fig. 6A, a fast [Ca^2+^]_i_ influx was observed after fMLF stimulation. In our study, apremilast did not diminish the peak [Ca^2+^]_i_ in fMLF-activated neutrophils, but it inhibited the time needed for [Ca^2+^]_i_ to decline to half of its peak (*t*_*1/2*_). The PKA inhibitor, H89, successfully reversed the inhibitory effect on *t*_*1/2*_ (Fig. 6).

### Inhibition of ERK and c-Jun N-terminal kinase (JNK) activation

**Fig. 7:**
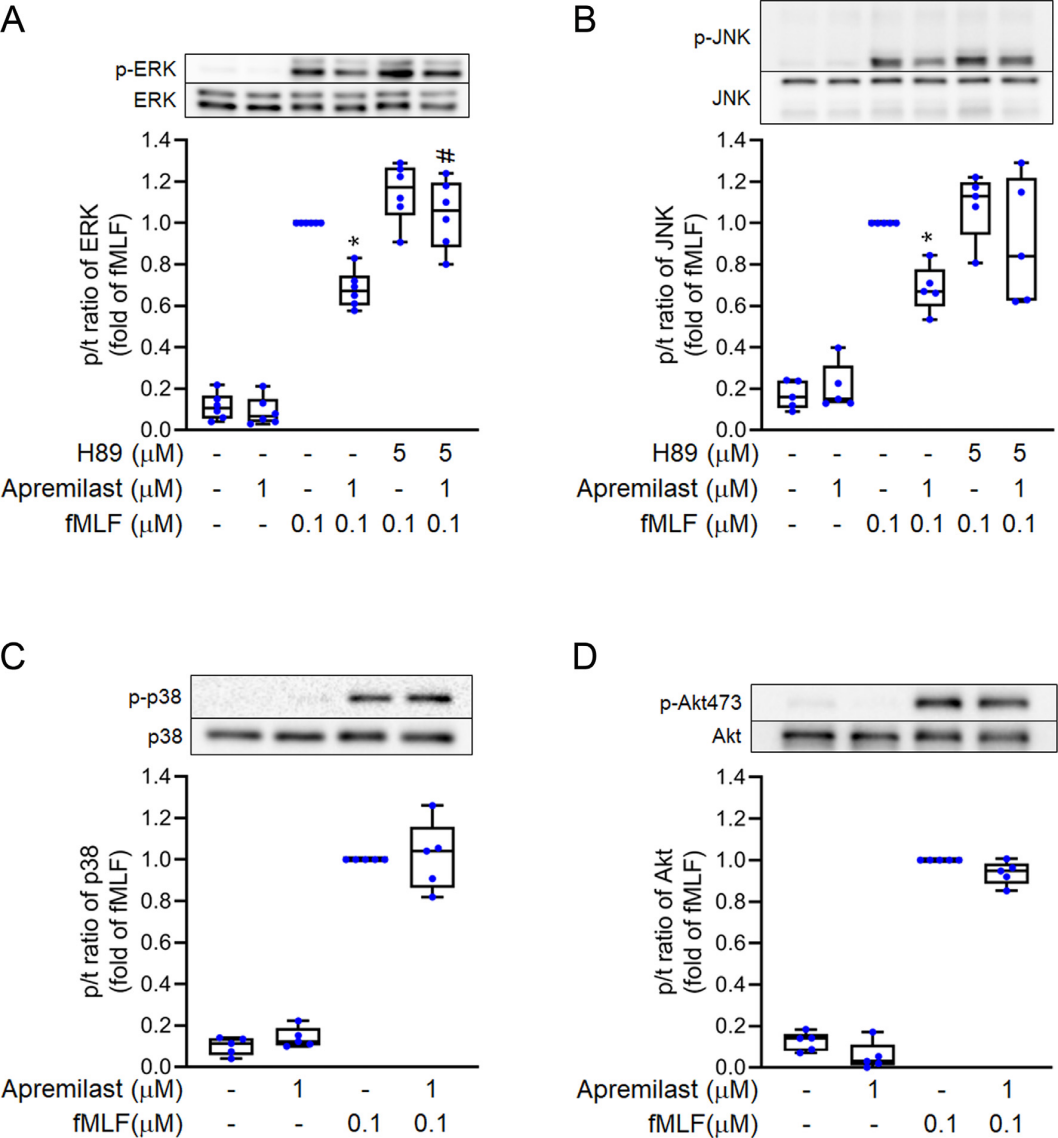
**Apremilast inhibits the phosphorylation of ERK and c-Jun N-terminal kinase (JNK) mediated by PKA signaling**. Neutrophils were pre-treated with N-[2-[[3-(4-bromophenyl)-2-propenyl] amino]ethyl]-5-isoquinolines-ulfonamide (H89) (5 μM) for 5 min and then co-incubated with apremilast (1 μM) at 37 °C. After being induced by *N*-formyl-l-methionyl-l-leucyl-l-phenylalanine (fMLF) (0.1 μM) for 30 s, phosphorylation of (A) ERK (B) JNK, (C) p38 MAPK, and (D) protein kinase B (Akt) proteins was assayed by immunoblot assays. All values are normalised to the reference control and are represented as mean ± SEM from independent experiments (n = 5–6). ∗*p* < 0.05 compared with the control value (fMLF only). ^#^*p* < 0.05 compared with its corresponding group (without H89 pre-treatment).

The phosphorylation of ERK, JNK, p38 MAPK, and Akt mediates formyl peptide receptor 1-induced neutrophil responses [32]. As shown in Fig. 7, fMLF induced the rapid phosphorylation of ERK, JNK, p38 MAPK, and Akt in human neutrophils. Apremilast significantly attenuated the upregulated phosphorylation of ERK and JNK but not of p38 MAPK and Akt. In addition, the inhibitory effects on ERK and JNK signals were reversed by H89 treatment (Fig. 7).

### LPS-induced acute respiratory distress syndrome (ARDS) mouse model

**Fig. 8:**
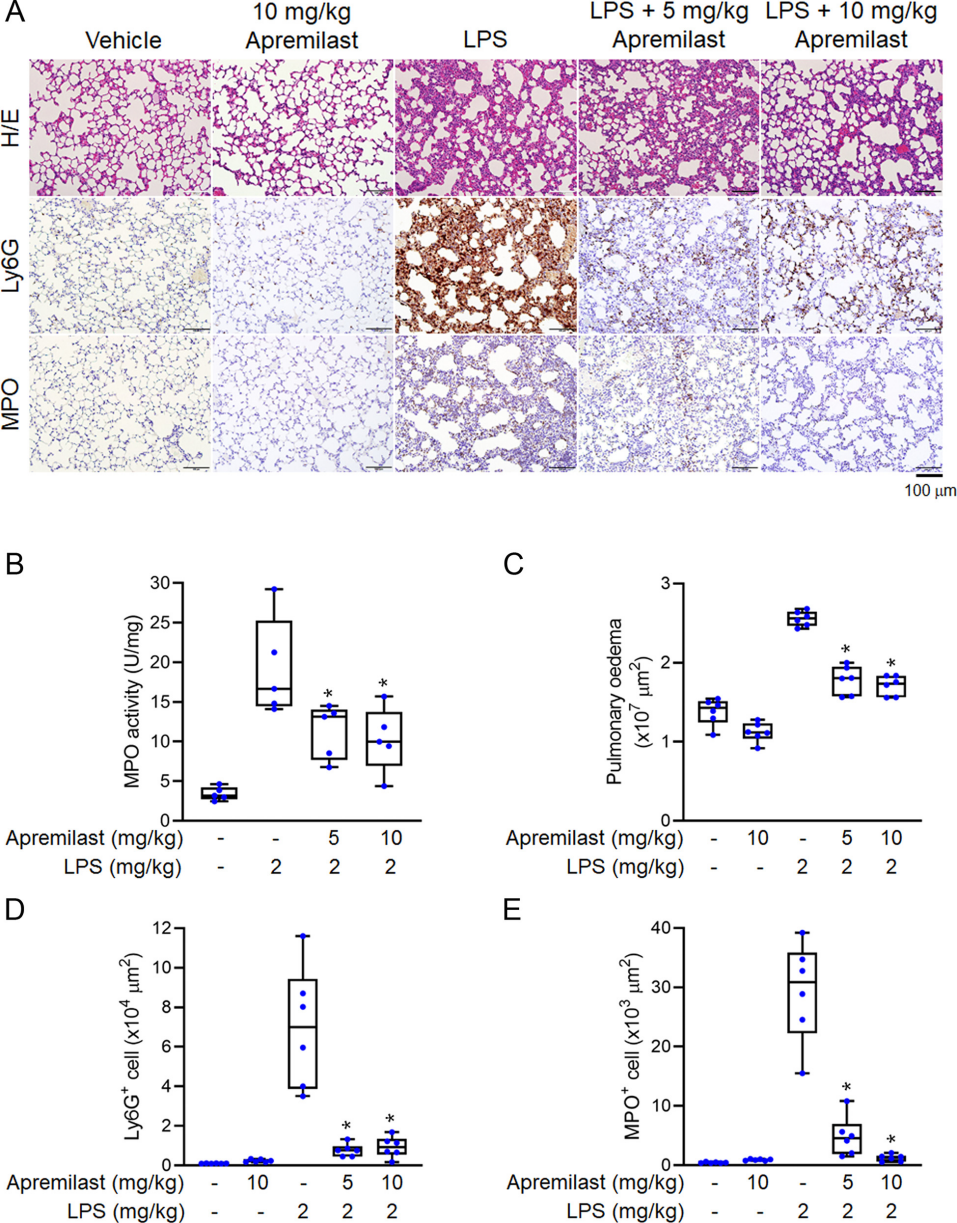
**The effect of intravenous apremilast on LPS-induced acute respiratory distress syndrome (ARDS) in mice**. (A) The histology of mouse lungs was examined by haematoxylin–eosin (H/E) staining, and immunohistochemistry using anti-Ly6G, anti-MPO and anti-4-HNE antibodies. Quantitative analysis of (B) H/E-stained tissues, and Ly6G^+^, MPO^+^ and HNE^+^ cells (C) MPO activity of mouse lungs. Data were represented as mean ± SEM from independent experiments (n = 6 per group). ∗*p* < 0.05 compared with the control value.

A mouse model of LPS-induced ARDS was used to evaluate the *in vivo* efficacy of apremilast. Histopathologic examinations of lung damage in the four groups are illustrated in Fig. 8A. Intratracheal LPS instillation resulted in an increase in MPO activity in the lungs, and the upregulation was reduced by intraperitoneal administration of apremilast (Fig. 8C). The H/E-stained lung sections of LPS-induced mice with ARDS-like symptoms revealed typical alveolar damage features, including interstitial oedema, alveolar oedema and haemorrhage, alveolar collapse, immune cell infiltration, and pulmonary congestion (Fig. 8A and C). Apremilast (5 and 10 mg kg^−1^) treatment significantly diminished lung neutrophil infiltration, MPO activity, alveolar damage, and oedematous changes in LPS-induced ARDS. MPO, Ly6G and HNE are representative biomarkers of neutrophil infiltration. As depicted in Fig. 8B, a predominant increase in immunofluorescence of MPO, Ly6G and HNE expression was observed in IHC examinations of LPS-induced ARDS. Neutrophil recruitment was effectively mitigated by apremilast treatment. In addition to therapy for psoriasis, our results suggest the use of apremilast as a new therapeutic choice for ARDS.

## Discussion

Previous studies have not investigated the anti-inflammatory effects of apremilast in the regulation of neutrophil activation and in its off-label application for the treatment of ARDS. Our present study showed the therapeutic effect of apremilast in the treatment of ARDS and in the suppression of neutrophil-mediated inflammatory responses. The pathogenesis and severity of ARDS are closely related to neutrophil activation [6]. Our study reveals that apremilast can essentially regulate oxidative stress and chemotaxis in activated neutrophils, and it demonstrates an alternative application for this medication, other than its use in treating psoriasis. Viral or bacterial invasion may induce an immunologic storm and the subsequent release of inflammatory mediators, resulting in severe lung injury or ARDS [33]. Currently, limited effective drugs are available for the treatment of ARDS. Current reports on steroid usage for treating ARDS-induced inflammation demonstrate poor definitive evidence of improved mortality [34]. Our study evaluated the therapeutic effects of apremilast on ARDS and the transduction pathway mediated in neutrophils. Apremilast selectively inhibited PDE4 activity via cAMP/PKA signaling to mitigate superoxide anion release, ROS production, CD11b expression, and neutrophil adhesion in activated neutrophils.

Oxidative stress is greatly induced in the regulation of inflammatory reactions in neutrophilic diseases [35]. Overabundance and dysregulation of oxidative stress contribute to the pathogenesis of ARDS. Superoxide anions and ROS increases are representative of oxidative stress in neutrophils. Neutrophil activation promotes respiratory burst reactions to generate a considerable amount of superoxide anions and of ROS derived from these anions via nicotinamide adenine dinucleotide phosphate (NADPH) oxidase activation [36]. Our results revealed that apremilast reduced superoxide anion and ROS production in fMLF-activated, but not in PMA-activated neutrophils (Fig. 1). In addition, assays for evaluating free radical scavenging abilities and LDH release were conducted to determine whether the inhibitory effects of apremilast on oxidative stress are mediated through its antioxidant activity or cytotoxicity. Apremilast showed no antioxidant ability to scavenge superoxide anions and free radicals in cell-free systems and was non-cytotoxic to neutrophils (Supplementary Fig. 1). The effects of apremilast against oxidative stress in neutrophils are attributed to upstream signaling pathway regulation rather than to direct superoxide scavenging or cytotoxicity.

Chemotaxis is a drawing force that recruits neutrophils to inflammation sites. Upon infection, circulating neutrophils infiltrate and adhere to the endothelium and then migrate into inflamed lungs. We evaluated human neutrophil chemotactic responses, including integrin expression on the cell membrane, and the adherence of neutrophils to the surface of endothelial cells. Our data showed that apremilast reduced the expression of the adhesion molecule, CD11b, and cell adhesion ability in fMLF-activated neutrophils (Fig. 2). The results confirmed that apremilast inhibits neutrophil activation by inhibiting oxidative stress, attenuating CD11b expression, and restricting cell adhesion. Therefore, apremilast shows potential as an adjuvant therapy for neutrophilic pathogenesis.

cAMP is an important second messenger that regulates cell function. In neutrophil activation, intracellular cAMP plays a role in negative regulation [37]. Upon induction by fMLF in neutrophils, apremilast significantly increased the level of intracellular cAMP and activity of PKA (Fig. 3A and B), which was responsible for the suppression of inflammatory responses. Previous studies have shown that in neutrophils, cAMP-specific PDEs are major enzymes rather than cGMP-specific PDEs [30]. Apremilast reduced the activities of cAMP-specific PDEs, but not that of ACs (Fig. 3C and D). cAMP-specific PDEs in neutrophils include PDE3, PDE4, and PDE7 subtypes, and the PDE4 family is the most abundant group among them. Apremilast showed selectivity for PDE4 inhibition but did not affect PDE3 or PDE7 (Fig. 4). We have provided evidence that apremilast predominantly inhibited all PDE4 activities of the A, B, C, and D isoforms. Our results suggest that apremilast inhibits intracellular PDE4 activity to induce an increase in cAMP and PKA activity in neutrophils. Our study further confirmed that the inhibitory effects of apremilast on oxidative stress, CD11b expression, and adhesion were mediated through PKA signaling (Fig. 5).

The second messenger, [Ca^2+^]_i_, is a critical target for inflammatory reactions that activate neutrophils [36]. Upon treating neutrophils with fMLF [Ca^2+^]_i_ rapidly increased. Apremilast enhanced the rate of [Ca^2+^]_i_ decline (*t*_*1/2*_) in fMLF-activated neutrophils (Fig. 6). This decrease in *t*_*1/2*_ in activated neutrophils was abrogated by the PKA inhibitor, H89. ERK and JNK signaling pathways also contribute to neutrophil activation [24,27]. Our results showed that apremilast impeded fMLF-induced phosphorylation of ERK and JNK, and the inhibitory effects were reversed by the PKA inhibitor (Fig. 7). The augmentation of Ca^2+^ mobilisation and phosphorylation of ERK and JNK is involved in neutrophil activation and facilitates oxidative stress upregulation. Excessive oxidative stress produced by activated neutrophils results in the damage of lungs in ARDS.

The present study evaluated the therapeutic effects of apremilast in the treatment of ARDS. The LPS-induced ARDS model was adopted, and the results revealed that the severity of lung damage is correlated with the infiltration of neutrophils [38,39]. Exaggerated neutrophil activation in inflammation can induce lung tissue injury and disrupt the alveolar-capillary barrier to increase permeability by increasing oxidative stress, elastase, and proinflammatory mediators. During the *in vivo* study of the ARDS model, alveolar morphology and neutrophil infiltration indicated by anti-Ly6G, anti-MPO and anti-4-HNE antibodies were examined by H/E and IHC staining. MPO, located in neutrophil granules, is an essential marker for neutrophil infiltration for evaluating the development of acute lung injury. Apremilast attenuated MPO activity, alveolar endothelium disruption, and wall oedema in ARDS-like pulmonary tissues (Fig. 8). Microscopic analysis of lung tissues by IHC examinations also confirmed significant inhibition of neutrophil infiltration in mice that were administered apremilast. Our study verified that apremilast reduced neutrophilic oxidative stress to reduce the severity of pulmonary inflammation in ARDS. Apremilast can alleviate LPS-induced ARDS-like injuries, and may serve as an adjunct therapy for the treatment of ARDS related to over-activated neutrophils. In the future, apremilast may have an off-label use for the treatment of ARDS via its restrictive effects on neutrophil-dominated inflammation.

**At a glance commentary**

**Scientific background on the subject**

The complication and mortality rate of patients with acute respiratory distress syndrome (ARDS) is remain high and poor management. Effective treatment is elusive and limited. The pathogenesis and severity of ARDS may attribute to the dysregulation of oxidative stress and neutrophil recruitment.

**What this study adds to the field**

Apremilast is a newly selective PDE4 inhibitor to treat the moderate to severe plaque psoriasis and psoriatic arthritis. Our study reveals the novelty anti-inflammatory effects of apremilast on activated human neutrophils and unapproved application for treating ARDS. Apremilast inhibits inflammatory responses after neutrophil activation via cyclic adenosine monophosphate(cAMP)/PKAdependent inhibition of ERK and c-Jun N-terminal kinase (JNK) activation. Apremilast can alleviate lipopolysaccharide-induced ARDS. It is expected that apremilast has potential of alternative off-label use for treating ARDS.

## Conflicts of Interest

None.
