# Supplementary material for: Apremilast ameliorates acute respiratory distress syndrome by inhibiting neutrophil-induced oxidative stress
Source: Biomed J. 2022 Sep 11;46(4):100560. doi: 10.1016/j.bj.2022.09.001 (PMC10345255; doi:10.1016/j.bj.2022.09.001)
Supplement: Multimedia component 1 [file mmc1.docx]

**Apremilast ameliorates acute respiratory distress syndrome by** **inhibiting neutrophil-induced oxidative stress**

**
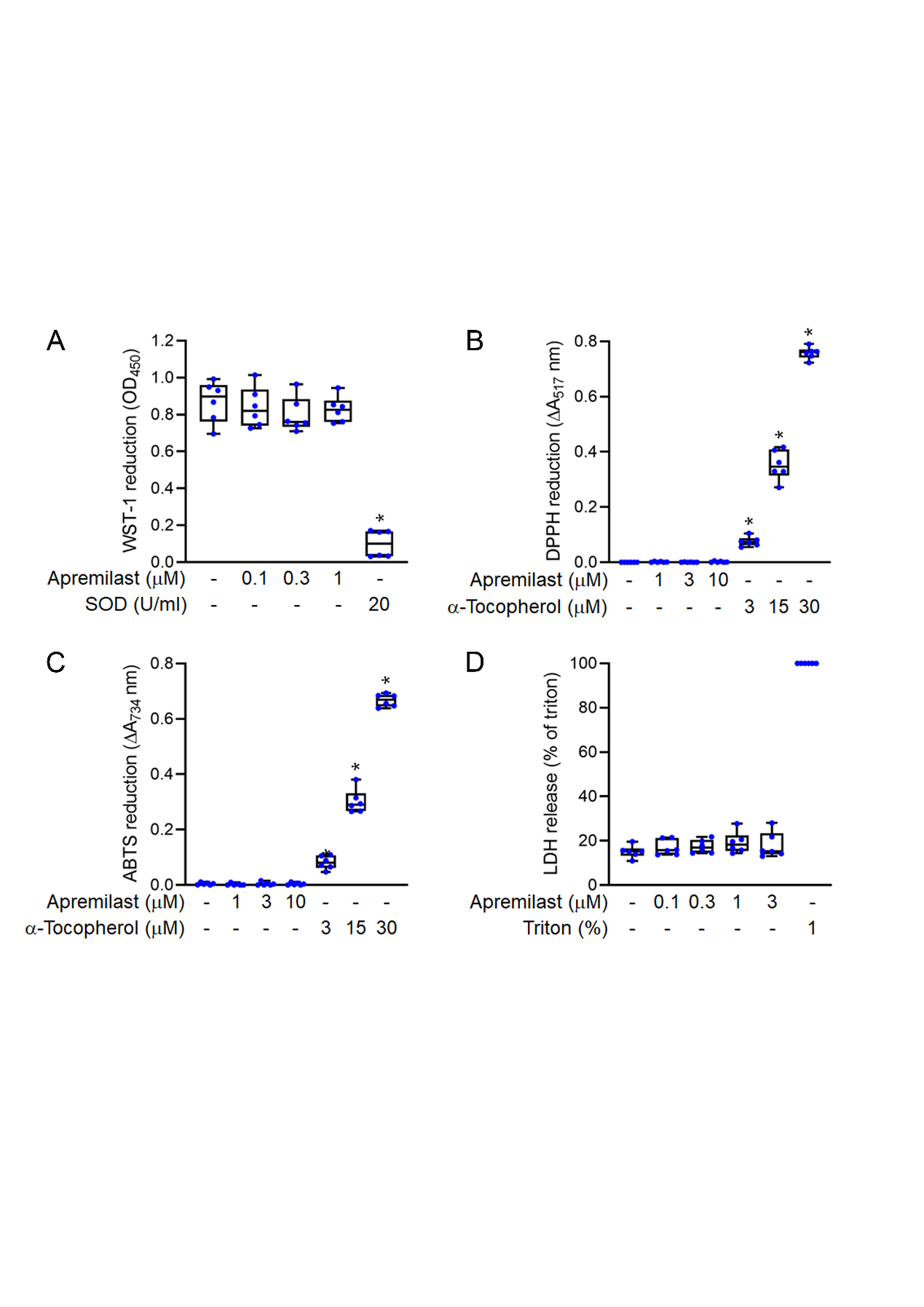
**

**Supplementary Fig. 1. Apremilast has no scavenging effects for free radicals and no cytotoxicity.** (A) Xanthine oxidase was incubated with DMSO, apremilast (0.1, 0.3, and 1 μM), or SOD (20 U mL^−1^) for 3 min. After administration of xanthine (0.1 mM) for 10 min, xanthine/xanthine oxidase-induced superoxide anions resulted in the decrease of the WST-1 level. The antioxidant effects of apremilast were measured using a spectrophotometer at 450 nm (n = 6). After treatment with DMSO, apremilast (1, 3, and 10 μM), or α-tocopherol (3, 15, and 30 μM), the decreases of (B) DPPH and (C) ABTS were determined using a spectrophotometer at 517 and 734 nm, respectively (n = 6). (D) Neutrophils were pre-treated with DMSO or apremilast (0.1, 0.3, and 1 μM) for 15 min. LDH release was detected for cytotoxicity using an ELISA kit (n = 6). Data represent the mean ± SEM from independent experiments. **p* < 0.05 compared with the control value.
